# Exploring the mechanism of baicalein on choroid melanoma based on network pharmacology, transcriptomics and experimental verification

**DOI:** 10.3389/fphar.2026.1787798

**Published:** 2026-04-08

**Authors:** Lili Wu, Yue Li, Jiancheng Gan, Anan Wang, Boyuan Zhang, Jinhai Yu, Qihua Xu, Hongfei Liao

**Affiliations:** 1 Jiangxi Provincial Key Laboratory for Ophthalmology, Nanchang, Jiangxi Clinical Research Center for Ophthalmic Disease, Jiangxi Research Institute of Ophthalmology and Visual Science, Jiangxi Medical College, Affiliated Eye Hospital of Nanchang University, Nanchang, China; 2 Department of Ophthalmology, The First Affiliated Hospital, Fujian Medical University, Fuzhou, China

**Keywords:** baicalein, choroidal melanoma, network pharmacology, PI3K/Akt/mTOR signaling pathway, transcriptomics

## Abstract

**Objective:**

Baicalein (BAI), a flavonoid from Scutellaria baicalensis, exhibits therapeutic potential across inflammation, viral infections, and cancers. Yet, its role in choroidal melanoma (CM), the most common intraocular malignancy, remains poorly understood. The aim of this study was to investigate BAI’s anti-CM effects and underlying mechanisms by integrating network pharmacology, transcriptomic analyses, and experimental validation.

**Methods:**

Network pharmacology, molecular docking and transcriptomic analysis were used to identify potential targets and signaling pathways. The anti-tumor effect of baicalein was verified through combined intracellular and extracellular experiments. Western blotting was used to analyse the effects of BAI and IGF-1 co-treatment on the phosphorylation level of the pathway and its downstream cycling, apoptosis and migration related proteins.

**Results:**

Integrated analyses implicated the inhibitory effect of BAI on choroidal melanoma may be related to the cell cycle, apoptosis and critical signaling pathways, particularly the PI3K/AKT/mTOR axis. *In vitro* experiments have proved that BAI can inhibit the proliferation and migration of C918 and OCM-1 cells, induce cell cycle arrest, and simultaneously trigger mitochondrial-mediated apoptosis and oxidative stress injury. Similar results were observed in the allograft tumor model of C918. IGF-1 pretreatment attenuated BAI’s effects, confirming pathway dependency.

**Conclusion:**

Our study demonstrates for the first time that BAI can inhibit CM malignant progression by modulating the PI3K/AKT/mTOR signaling pathway. This study suggests that BAI may be used as a promising anti-tumor agent for further treatment of CM.

## Graphical Abstract

**Figure:**
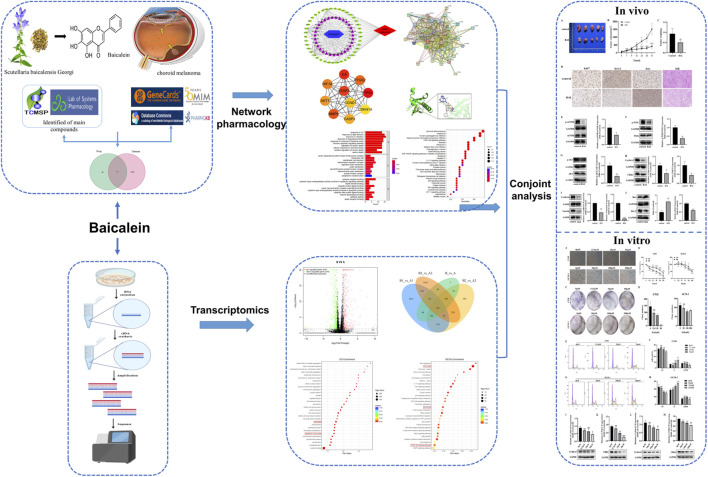

## 1 Introduction

Choroidal melanoma (CM) represents the most common and aggressive primary intraocular malignant tumor in adults (Chattopadhyay et al., 2016; Rai et al., 2017). Owing to the highly vascularized nature of the choroid and the absence of lymphoid lymphatic drainage, the intraocular microenvironment provides a conducive niche for hematogenous metastasis of CM cells to distant organs (Chen et al., 2022; Onken et al., 2014). Clinically, 40%–50% of CM patients develop metastasis, with the liver being the most frequently affected site (up to 90% of cases). The prognosis for metastatic CM is dismal, with a median survival of approximately 6 months (Beasley et al., 2022; Ragusa et al., 2015; Saldanha et al., 2025). Current therapeutic strategies for choroidal melanoma, such as ocular extraction, local excision, chemotherapy, immunotherapy, and targeted therapy, have resulted in better control of the primary tumor to a certain extent, but the prognosis of the patients is not well improved and the efficacy of the treatment is much less than that of cutaneous melanoma (Jager et al., 2020; Li et al., 2023). Therefore, there is an urgent need for a new therapeutic strategy to enhance the clinical management of CM.

Natural products derived from traditional Chinese medicine (TCM) have garnered significant attention for their antitumor properties (Li et al., 2025; Wen et al., 2025; Yu et al., 2024). Previous studies conducted by our team have shown that these TCM components can exhibit potent anticancer effects by inducing apoptosis, inhibiting cell proliferation, metastasis and vasculogenic mimicry (Shi et al., 2021a; Shi et al., 2021b). Baicalein, a natural flavonoid extracted from the roots of Scutellaria baicalensis, has been shown to possess a variety of pharmacological activities such as anti-inflammatory, antioxidant, anti-tumor and neuroprotective (Dinda et al., 2017; Yu et al., 2017a). Accumulating evidence indicates that baicalin possesses potent cytotoxicity against various malignancies including prostate cancer (Gioti and Tenta, 2015), osteosarcoma (Zhang et al., 2013), breast cancer (Chung et al., 2015; Yan et al., 2018), hepatocellular carcinoma and non-small cell lung cancer (Li et al., 2021; Qi et al., 2023; Su et al., 2018; Yang et al., 2022). Based on these findings, we hypothesized that similar anticancer effects of baicalein exist in choroidal melanoma. However, no studies have been conducted to elucidate the potential molecular mechanisms underlying the anticancer activity of baicalein in choroidal melanoma.

The PI3K/AKT/mTOR signaling pathway is one of the most important intracellular signaling pathways, and dysregulation of this pathway is closely related to biological processes such as growth, motility, survival, metabolism and angiogenesis of tumor cells (Jiang et al., 2025; Katso et al., 2001; Martini et al., 2014). Both our preliminary data and existing literature have identified the PI3K/AKT pathway as a crucial target for TCM-derived agents in the suppression of CM (Sun et al., 2025a; Yan et al., 2020). The role of baicalein in inhibiting tumors by targeting the PI3K/AKT/mTOR axis has been extensively validated in other cancer types (Qiao et al., 2022). Therefore, intervening in this pathway is highly likely to be the core mechanism by which baicalein treats CM.

Network pharmacology, as an emerging interdisciplinary research field deeply integrating bioinformatics and systems biology, has opened new avenues for dissecting the intricate interactions between drugs and diseases (Liu et al., 2024). This field is in line with the overall philosophy of TCM and is commonly used to predict potential bioactive components and major functional targets of TCM and to investigate the underlying mechanisms of these drug operations (Li and Xiao, 2025; Zhang et al., 2025). Transcriptomic technology has achieved a technological breakthrough in analyzing the laws of disease development from the gene expression dimension by constructing organism-wide transcript expression profiles (Stoeckius et al., 2017). This technology provides solid technical support for the in-depth understanding of the molecular mechanism of disease development and the discovery of novel biomarkers. Combining network pharmacology with transcriptome analysis represents an effective approach to identify active compounds and mechanisms of action of traditional herbal formulations, which is becoming increasingly common in TCM research (Zhu et al., 2021).

In this study, we used an integrative strategy that incorporates network pharmacology, transcriptomics, and molecular docking techniques to elucidate the mechanism of action and molecular targets of BAI against choroidal melanoma. The predictive findings were subsequently validated through *in vitro* cellular experiments and *in vivo* animal models. These comprehensive analyses and experimental validations provide a strong scientific basis for future clinical applications.

## 2 Materials and methods

### 2.1 Network pharmacological analysis

#### 2.1.1 Screening of effective active ingredients and action targets of scutellaria baicalensis georgi

Scutellaria baicalensis was searched in the Traditional Chinese Medicine systematic pharmacological database and analytical platform (TCMSP) (https://old.tcmsp-e.com/tcmsp.php), and OB ≥ 30% and DL ≥ 0.18 were used as the screening screening criteria to identify the active ingredients and related targets of Scutellaria baicalensis Georgi (SBG). Then, the drug target genes were characterized by UniProt database (https://www.uniprot.org/). (Han et al., 2022; Huang et al., 2025).

#### 2.1.2 Acquisition of potential therapeutic target genes of uveal melanoma

Using “uveal melanoma” as the keyword, the GeneCards (https://www.genecards.org/), OMIM(https://www.omim.org/), PharmGkb (https://www.pharmgkb.org/) and Disgenet (https://www.disgenet.org) databases were searched to obtain the genes associated with uveal melanoma (UM) (Tan et al., 2025).

#### 2.1.3 Obtaining intersection target genes of SBG and UM

The Venn diagram package of R language 4.2.1 was used to cross the target genes of the drug and the target genes of uveal melanoma, and the intersection genes of Scutellaria anti-UM were obtained (Tu et al., 2021).

#### 2.1.4 Construction of the SBG-compound-target-uveal melanoma network

By importing the complex relationships between SBG, active components, UM and common targets into Cytoscape 3.9.1 software, we constructed a drug-component-target-disease network and identified the major components based on topological parameters (Pan et al., 2022).

#### 2.1.5 Protein-protein interaction network construction and functional enrichment analysis

Firstly, the STRING database was used to construct the Protein-protein interaction (PPI) network, and finally the PPI network data was output in CSV format. The screening condition of the organism is set to “*Homo sapiens*”, and the required interaction score ≥0.7” (Wang et al., 2025; Wu et al., 2024). Cytoscape 3.9.1 was then used to visualize and topologically analyze the PPI network structure. We selected the target nodes with degree values, betweenness centrality and closeness centrality, which were higher than the corresponding median values in PPI network, and predicted the probable core targets in 10 for further study (Sun et al., 2025b; Zhou et al., 2023). Functional enrichment analysis was performed using the clusterProfiler package in R language for both Gene Ontology (GO) and Kyoto Encyclopedia of Genes and Genomes (KEGG) enrichment analyses (Lin et al., 2023).

### 2.2 Molecular docking

The 2D structure of the small molecule ligand was obtained from the PubChem database (https://pubchem.ncbi.nlm.nih.gov/), and the 2D structure was converted into a 3D structure and into a file of mol2 using Chem3D software. The protein ID corresponding to the core gene was found using uniprot, then the 3D structure of the protein (1H10) was obtained from the PDB database (https://www.rcsb.org/), and the protein was dehydrated, stripped of small-molecule ligands, and hydrogenated using the PyMOL software (Hou et al., 2022). The core gene proteins and their major components were converted into PDBQT format using AutoDock Tools 1.5.6 software, and the predicted active pocket information was converted into PDBQT format using the deepsite website. Finally, molecular docking was performed using AutoDock vina and the results were visualized using PyMOL software (Sun et al., 2025b).

### 2.3 Transcriptome RNA sequencing

C918 cell cultures were collected 48 h following baicalein treatment administration. Total RNA extraction was performed on untreated control samples (A1, A2, A3) and baicalein-exposed experimental samples (B1, B2, B3) using Trizol reagent provided by Sangon Biotech (Shanghai, China). RNA quality control involved assessment of purity and concentration via nanodrop spectrophotometry and bioanalyzer analysis to verify compliance with downstream sequencing standards. Sequencing services were contracted to Sangon Biotech Co., Ltd., with the resulting datasets stored in standardized Sanger FASTQ compression format. Differential expression analysis was performed using bioinformatics algorithms, applying stringent filtering criteria (FDR<0.05 and |log2FoldChange|>1) to identify statistically significant differentially expressed genes (DEGs). Results were visualized using volcano plots and Venn diagram representations to highlight unique and overlapping gene expression profiles. Functional enrichment analyses were subsequently performed on DEGs using GO and KEGG databases to elucidate the molecular pathways modulated by baicalein in choroidal melanoma cells.

### 2.4 Chemical compounds

The powder of baicalein (HLPC≥ 98%, no. SB8010) was obtained from Solarbio Biotechnology Co., Ltd. (Beijing, China) and dissolved in dimethyl sulfoxide (DMSO; Roche, Switzerland). A 200 mM stock solution were prepared and stored in −20 °C for the experiments and thawed at room temperature before use.

### 2.5 Cell culture and treatment

Human choroidal melanoma cell lines C918 and OCM-1 cells were purchased from Shenzhen Hitouch Technology Co. Ltd. and Shanghai Fuheng Biotechnology Co. Ltd, respectively. C918 and OCM-1 cells were cultured in RPMI-1640 and high-sugar Dulbecco’s Modified Eagle’s Medium (DMEM) containing 1% penicillin/streptomycin and 10% fetal bovine serum respectively (BI, Israel). All cells were cultured at 37 °C in a humidified atmosphere containing 5% CO2.

Two different grouping strategies were used to treat two types of cells in this study: (I) To evaluate the effects of BAI on C918 and OCM-1 cells in terms of proliferation, apoptosis, migration, and epithelial-mesenchymal transition (EMT), we set different BAI concentrations and treatment times based on the results of cytotoxicity tests. Specifically, C918 cells were divided into four groups and treated with 0, 12.5, 25, and 50 μM BAI for 48 h, while OCM-1 cells were divided into another four groups and treated with 0, 50, 100, and 200 μM BAI for the same length of time.

(II) To further explore the role of PI3K/AKT/mTOR signaling axis in BAI anti-choroidal melanoma, C918 cells were divided into: 0 μM BAI group, 50 μM BAI, 0 μMBAI + IGF-1 group and 50 μM BAI + IGF-1 group. OCM-1 cells were divided into: 0 μM BAI group, 200 μM BAI, 0 μM BAI + IGF-1 group and 200 μM BAI + IGF-1 group. Before the experiment, all were pretreated with 200 ng/mL of IGF-1 for 6 h using both to activate the PI3K/AKT/mTOR signaling axis.

### 2.6 Cell viability assay

The effect of BAI on cell viability was determined by CCK-8 assay (YeaSen, Shanghai, China). Briefly, When the cell confluence reached 80%–90%, C918 (4000 cells/well) and OCM-1 (6,000 cells/well) cells were digested and transferred to 96-well plates for incubation, and treated with different concentrations of baicalein (0, 12.5, 25, 50, 100, and 200 μM) for 24 and 48 h. After that, 100 ul of fresh medium containing 10% CCK-8 was added to each well. After 2 h of incubation, the cell viability was measured at an absorbance wavelength of 450 nm using an enzyme marker (Thermo Fisher Scientific, United States), and a dose-effect curve was fitted using GraphPad Prism 8.0 software.

### 2.7 Colony formation assay

C918 and OCM-1 cells were inoculated into 6-well plates at a density of 300 and 500 cells/well, respectively. The adherent cells were treated for 7–14 days according to the experimental grouping. When the number of clones in the control group exceeded 50, they were fixed with methanol for 20 min, and then stained by adding 0.25% crystal violet solution for 20 min, and the well plates were washed and dried, and then photographed and recorded.

### 2.8 Wound healing assay

Cells were cultured and incubated in 6-well plates for 24 h to achieve cell densities above 95%. The cells were scraped with a 200ul pipette tip, and after scraping, the cells were gently rinsed twice with PBS, after which fresh medium containing the indicated doses of baicalein was added to continue the incubation for 24 h and 48 h, and the scratches were observed with an inverted microscope (OLYMPUS, Japan) and pictures of the scratches were taken at 0 h, 24 h, and 48 h. The degree of closure was estimated as the ratio of the wound area relative to the initial area.

### 2.9 Transwell assay

C918 and OCM-1 cells were treated with different concentrations of baicalein, and the cell densities were adjusted to 2 × 10^5 cells/mL and 4 × 10^5 cells/mL with medium containing 0.1% FBS. Aliquots (200 μL) of cell suspension were pipetted into the upper transwell chambers, while the lower compartments contained 600 μL of chemoattractant medium (20% FBS). After 24 h of incubation, cells that had migrated through the membrane were fixed with methanol for 20 min and then stained with 0.1% crystal violet for 15 min. Five different fields of view were counted and tallied using ImageJ software.

### 2.10 Hoechst 33342 staining

C918 and OCM-1 cells (5 × 10^5 cells/well) were inoculated in six-well plates, and the cells were affixed to the wall and then treated with different concentrations of baicalein for 48 h. After being washed with PBS, 4% paraformaldehyde (ServiCebio, Wuhan, china) was added, and the cells were fixed for 20 min at room temperature. Subsequently, cells were stained with Hoechst 33,342 solution (Beyotime, Shanghai, China) for 10 min. Different areas of the stained cells were observed using an inverted fluorescence microscope (OLYMPUS, Japan).

### 2.11 Apoptosis assays

Apoptosis was evaluated utilizing an Annexin V-FITC/PI Apoptosis Assay Kit (YeaSen Biotech, Shanghai, China) following manufacturer-recommended procedures. 5 × 10^5^ cells, collected via EDTA-free trypsin digestion and centrifugation, were washed twice with PBS, resuspended in 100 μL binding buffer, and stained with 5 μL Annexin V-FITC and 10 μL PI for 10 min in darkness. Subsequent to labeling, 400 μL of additional binding buffer was incorporated to achieve optimal sample dilution for flow cytometric analysis, which was executed using a BD FACSCalibur system (Becton, Dickinson and Company, United States) with pre-set compensation.

### 2.12 Cell cycle analysis

Cell cycle distribution was analysed using a cell cycle detection kit (Beyotime, Shanghai, China). Cells (1 × 10^6) after different baicalein treatments were collected, washed with pre-cooled PBS, and fixed by adding 1 mL of 70% ethanol at 4 °C overnight. On the following day, after washing with pre-cooled PBS and centrifugation, 500ul of staining buffer containing PI/RNase A was added and incubated for 30 min at room temperature under dark conditions. Flow cytometry (BD Biosciences) and Flow Jo software were used for data acquisition and analysis.

### 2.13 Animal model and drug treatment

Male BALB/c nude mice aged 4 weeks were obtained from the Nanchang Experimental Animal Center (Jiangxi Province, China) for xenograft tumor model development. All *in vivo* procedures strictly adhered to ARVO guidelines for animal use in vision research and were approved by the Institutional Animal Care and Use Committee of Nanchang University under protocol NCULAE-20240605001. Matrix gel was mixed and diluted with serum-free medium in a 1:1 ratio for resuspending the digested C918 cell precipitates, and the cell concentration was adjusted to 5 × 10^6 cells/ml. Then 200 µL of C918 cells were inoculated into the posterior axilla of nude mice. When the tumor volume reached 100 mm^3^, all nude mice were randomly divided into two groups using the random number method: a vector control group receiving phosphate-buffered saline, and a treatment group by oral administration of 100 mg/kg baicalein per day. Tumor weight and volume were measured and recorded every 2 days. After 13 days of administration, mice were humanely euthanized using carbon dioxide (CO_2_) inhalation. The CO_2_ was introduced at a flow rate of 30% of the chamber volume per minute until the mice were confirmed to be deceased (Hartono et al., 2025; Yue et al., 2025). Tumors were removed and weighed and subjected to immunohistochemical staining, HE staining and protein immunoblot analysis.

### 2.14 Immunohistochemistry

Paraffin-embedded tissue sections were incubated at 65 °C for 1 h before being deparaffinised in xylene and then hydrated in alcohol. Subsequently, the sections were heated in citrate buffer for 10 min for antigenic repair of the tissue. After PBS washing, the sections were exposed to 3% hydrogen peroxide to block endogenous peroxidase activity. Non-specific protein binding was blocked through 1-h incubation with 5% bovine serum albumin (BSA) solution. Primary antibody incubation was conducted overnight at 4 °C using a panel of monoclonal antibodies, specifically: anti-Bcl-2 (HA721235, 1:1,000, HUABIO), anti-Bax (ET1603-34, 1:200, HUABIO), and anti-Ki-67 (GB111499, 1:1,000, Servicebio). On the following day, sections were washed with PBS and incubated with anti-rabbit IgG-HRP antibody (GB23303, 1:200, Servicebio) for 1 h at room temperature. Chromogenic development was accomplished using 3,3′-diaminobenzidine (DAB) substrate, followed by counterstaining with hematoxylin. Finally, tissue sections were dehydrated and sealed with neutral resin and observed under an inverted microscope (OLYMPUS, Japan).

### 2.15 HE staining

Paraffin-embedded tumor sections were initially dewaxed through oven incubation at 85 °C for 30 min. The specimens were immersed in xylene twice for 15 min, and then sequentially immersed in anhydrous ethanol, 95% ethanol, 80% ethanol, and 70% ethanol for 5 min each. Nuclear staining was executed using hematoxylin for 1 min after PBS rinsing. Subsequent differentiation involved running water rinsing (1 min), cytoplasmic staining with eosin (30 s), and final rinsing to remove excess dye. Subsequently, specimens were sequentially immersed in 70% ethanol for 10 s, 80% ethanol for 10 s, 95% ethanol for 30 s, anhydrous ethanol for 1 min, and xylene for 2 times for 1 min to dehydrate and clear. Air-dried sections were mounted with neutral resin and the results of the staining were observed under a microscope (OLYMPUS, Japan).

### 2.16 Western blot analysis

Treated cells or tissues were washed twice with ice-cold PBS and lysed using RIPA buffer (1:1:100) supplemented with protease/phosphatase inhibitors. Clarified lysates were obtained via centrifugation after sonication on ice. Protein extracts were resolved by SDS-PAGE and transferred to PVDF membranes (Millipore, Bedford, MA). Membranes were blocked with 5% non-fat milk (GC310001, Servicebio) for 2 h at room temperature, then incubated overnight at 4 °C with primary antibodies (Supplementary Material Table S1). After three TBST washes, membranes were incubated with species-specific secondary antibodies (SA00001-2/SA00001-1, Proteintech) for 1 h at room temperature. Chemiluminescent signals were detected using ECL reagent (K-12043-D10, Advansta) on a Syngene G: Box imager (Thermo Fisher Scientific). Grey scale values of the images were quantitatively analyzed using ImageJ software. Antibodies used in this study are listed in Supplementary Material Table S1.

### 2.17 Statistical analysis

Experimental results were expressed as mean ± SD of at least three independent experiments. All experimental data were processed and analyzed using Microsoft Excel and GraphPad Prism version 8.0 software. Comparisons between the two groups were made using t-tests, whereas one-way ANOVA was applied for the assessment of three or more cohorts. Statistical significance was defined as p < 0.05.

## 3 Results

### 3.1 Network pharmacology analysis predicts SBG targets and pathways affecting UM

**FIGURE 1:**
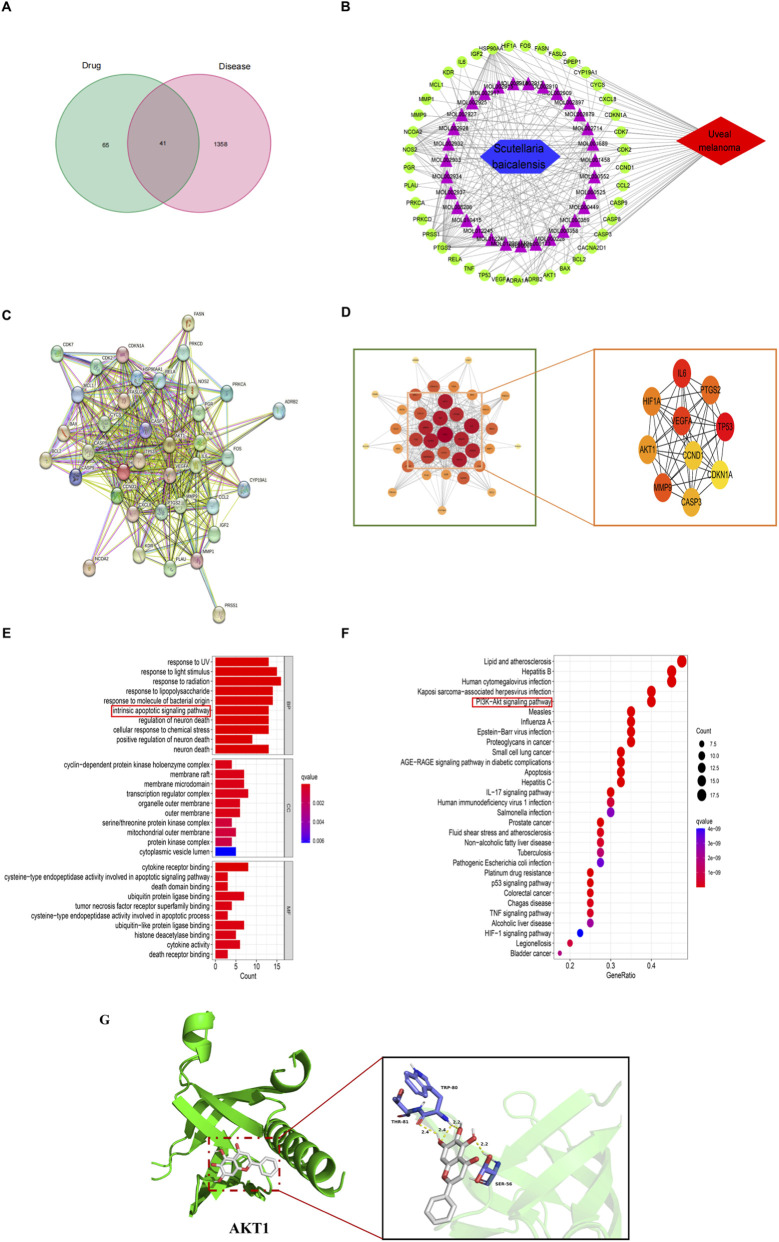
Network pharmacology analysis predicts SBG targets and pathways affecting UM. **(A)** Venn diagram of the intersection of SBG target genes with UM genes. **(B)** SBG-compound-target-uveal melanoma network diagram. **(C)** SBG anti-UM PPI network. **(D)** Screening of the top 10 core genes of the PPI network. **(E)** Barplot diagram of GO enrichment analyses. **(F)** Bubble diagram of the first 30 KEGG enrichment analyses. **(G)** Molecular docking results of BAI and AKT1.

To elucidate the potential therapeutic targets and pathways of SBG in the treatment of uveal melanoma, we conducted a network pharmacology analysis. The Venn diagram analysis revealed 41 overlapping targets between SBG and UM that could be potential therapeutic targets (Figure 1A). Subsequently, the SBG-compound-target-uveal melanoma network was constructed using Cytoscape software (Figure 1B). In order to further investigate the mechanism of these targets, 41 targets were entered into the STRING database to construct a PPI network, which was visualized by Cytoscape software and screened to identify the core genes with the top ten degree values (Figures 1C,D). As shown in Figure 1E, GO term analysis exhibited considerable enrichment in the intrinsic apoptotic signaling pathway. KEGG results showed that the genes were significantly enriched in the target pathway PI3K/AKT signaling pathway and molecular docking results also indicated that BAI interacted with AKT1 (Figures 1F,G). In short, network pharmacology analysis paves a crucial avenue for further exploring the fundamental mechanisms involved.

### 3.2 BAI inhibited the proliferation of CM cell lines and induced cell cycle S arrest

**FIGURE 2:**
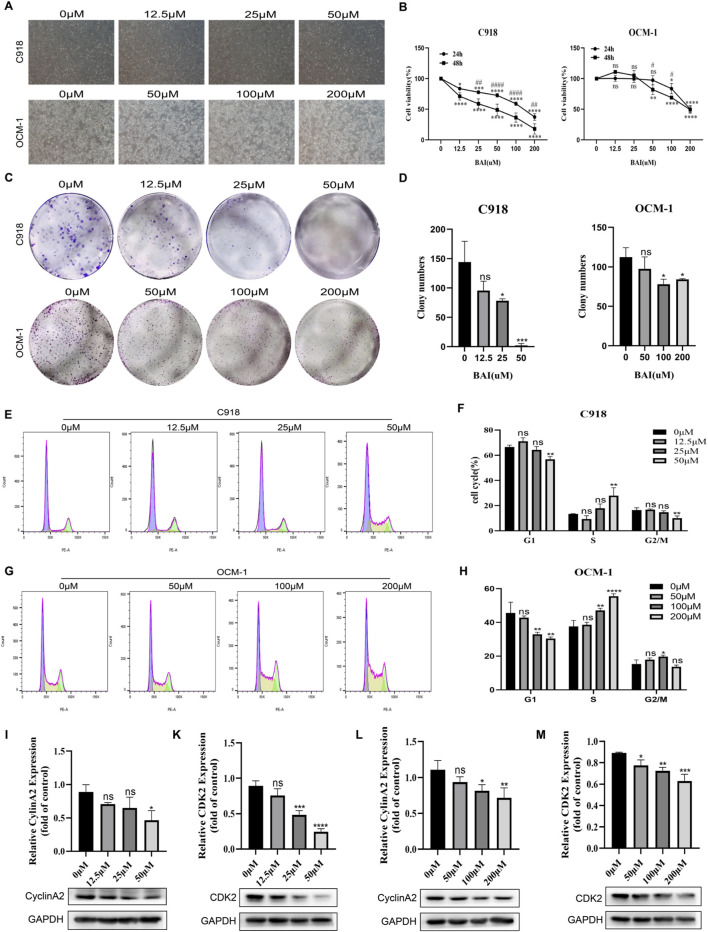
BAI inhibited the proliferation of CM cell lines and induced cell cycle S arrest. **(A)** Microscopic observation of the changes in cell density and morphology at different concentrations (0, 12.5, 25, 50, 100 and 200 μmol/L) of BAI after 48 h of treatment. **(B)** The effects of BAI on the viability of C918 and OCM-1 cells after incubation of cells with different concentrations of BAI for 24 and 48 were shown. **(C,D)** Distribution of clone formation in the control and dosing-treated groups. **(E–H)** PI staining analysis of cycle occupancy changes in C918 and OCM-1 cells. **(I–M)** Changes of cell cycle S-phase related proteins and their grey scale value analysis after different concentrations of BAI treated cells for 48 h. Data are expressed as mean ± SEM. n = 3, ns: not significant, *P < 0.05, **P < 0.01, ***P < 0.001, ****P < 0.0001 vs. control, #P < 0.05, ##P < 0.01, ####P < 0.0001 vs. BAI (24 h).

Based on the network pharmacology predictions, we hypothesized that BAI would exert antiproliferative effects on CM cells via targeting the PI3K/AKT pathway. To test this hypothesis, we evaluated the effect of BAI on the proliferative ability of C918 and OCM-1 using CCK-8 assays and clone formation experiments. Microscopic examination revealed a marked decrease in adherent cell populations following baicalein exposure (Figure 2A). CCK-8 results demonstrated concentration- and time-dependent suppression of C918 cell viability, whereas OCM-1 cells exhibited sensitivity only to concentrations exceeding 200 μmol/L at 24 h. Notably, extending treatment duration to 48 h induced dose-dependent viability reduction in OCM-1 cells even at 50 μmol/L, indicating enhanced sensitivity with prolonged exposure (Figure 2B). Clonogenic survival assays confirmed significantly impaired colony formation capacity in treated groups compared to controls (Figures 2C,D). Given the crucial role of the cell cycle in cell proliferation, PI-based flow cytometry was employed to evaluate cell cycle distribution post-treatment. Remarkably, 48 h baicalein exposure caused significant S phase accumulation accompanied by diminished G0/G1 and G2/M phase proportions relative to untreated controls (Figures 2E–H). To elucidate underlying mechanisms, immunoblottings were performed and revealed reduced expression of S phase-related proteins, including Cyclin A2 and CDK2, in treated cells (Figures 2I–M). All these results suggest that baicalein can exert antiproliferative effects on choroidal melanoma.

### 3.3 BAI triggers mitochondria-mediated apoptosis in CM cells

**FIGURE 3:**
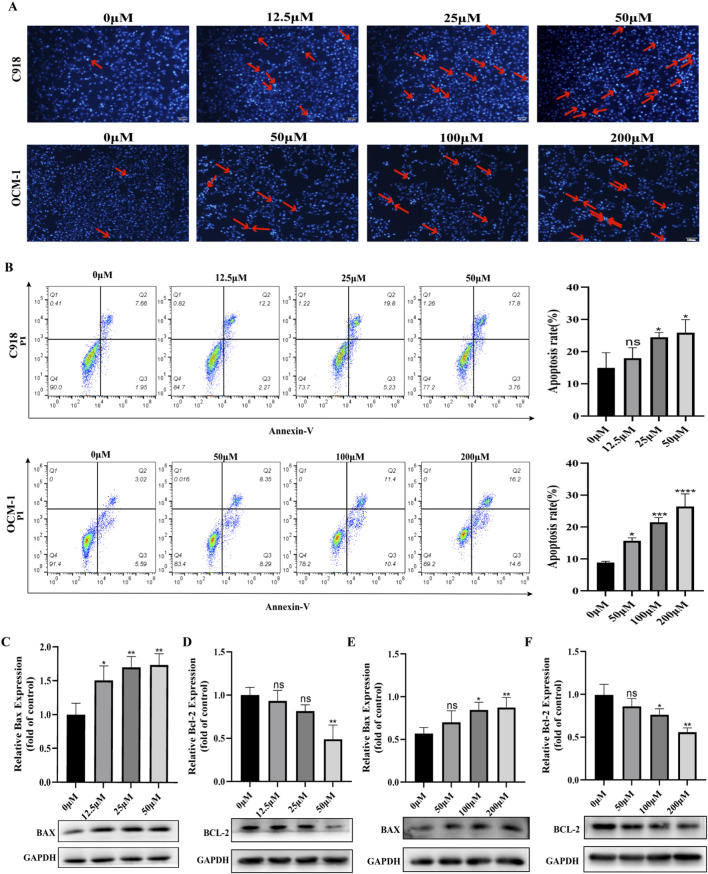
BAI triggers mitochondria-mediated apoptosis in CM cells. **(A)** Pictures of Hoechst33342 stained cells for apoptosis after 48 h of BAI treatment (the red arrow shows some of the apoptotic cells). **(B)** The apoptosis rate of each group was detected by flow cytometry with Annexin V-FITC/PI double staining. **(C–F)** Western blot detected the expression of apoptosis-related proteins Bcl-2 and Bax in C918 cells and OCM-1 cells. Data are expressed as mean ± SEM. n = 3, ns: not significant, *P < 0.05, **P < 0.01, ***P < 0.001, ****P < 0.0001 vs. control.

Given the enrichment of apoptotic pathways in our network pharmacology analysis, we further hypothesized that BAI induces apoptosis in CM cells. To test this, we conducted Hoechst 33,342 staining and Annexin V-FITC/PI flow cytometry analyses. In the untreated control groups of both cell lines, the nuclei exhibited faint and even staining, with apoptotic cells being scarce. Conversely, as the concentration of baicalein increased, there was a progressive rise in the proportion of cells displaying condensed nuclei and intense dark blue fluorescence, suggesting that apoptosis was induced by baicalein (Figure 3A). Flow cytometry analysis revealed that, in the negative control group, 9.61% of C918 cells and 8.61% of OCM-1 cells underwent spontaneous apoptosis. Upon treatment with 50 μmol/L of baicalein, the apoptotic rate in C918 cells rose to 21.56%, whereas in OCM-1 cells, a concentration of 200 μmol/L resulted in an apoptotic rate of 25.43% (Figure 3B). Although baicalein induced apoptosis in OCM-1 cells, its effect was moderate even at a high concentration. This suggests that while baicalein can trigger programmed cell death, its anti-proliferative effects may also be driven by other mechanisms such as cell cycle arrest or inhibition of migration, rather than solely relying on acute cytotoxicity. Subsequently, Western blot analysis was performed to assess the changes in the levels of apoptosis-related proteins. The results demonstrated a marked decrease in Bcl-2 levels in cells treated with baicalein, accompanied by a gradual upregulation of Bax levels (Figures 3C–F). In conclusion, these findings suggest that baicalein may induce apoptosis through the mitochondria-mediated apoptotic pathway.

### 3.4 BAI suppressed the migration and EMT of CM cells

**FIGURE 4:**
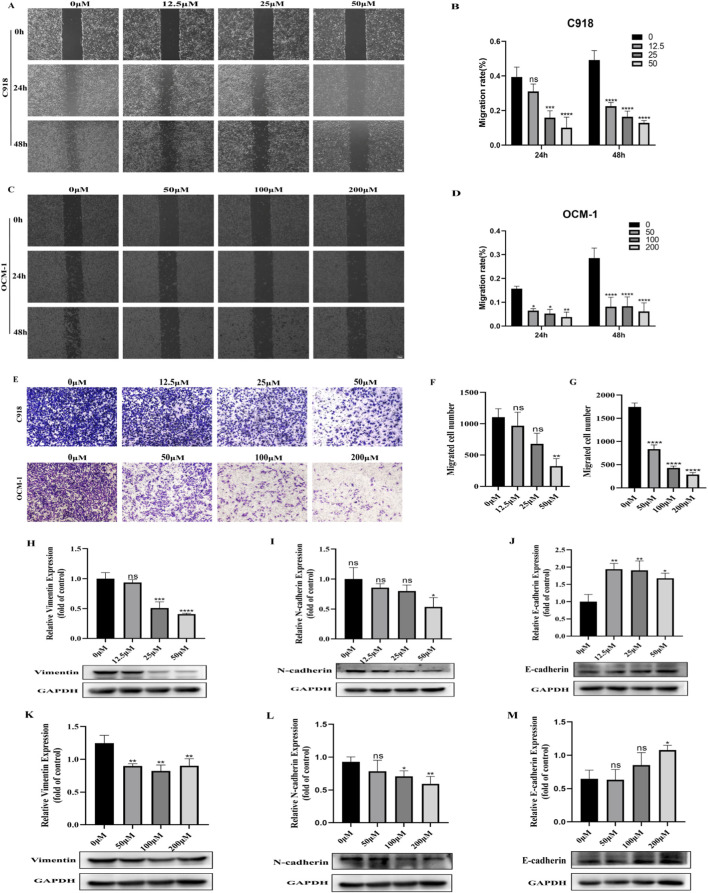
BAI suppressed the migration and EMT of CM cells. **(A–D)** Scratch assay to detect the migration distance and quantitative analysis of C918 and OCM-1 cells with different concentrations of BAI for 24 h and 48 h. **(E–G)** Transwell assay to detect the number of migrated cells after 48 h of BAI treatment and quantitative analysis. **(H–M)** Western blot to detect the expression changes of EMT-related proteins in C918 and OCM-1 cells and quantitative analysis. Data are expressed as mean ± SEM. n = 3, ns: not significant, *P < 0.05, **P < 0.01, ***P < 0.001, ****P < 0.0001 vs. control.

Given that cell migration and EMT are important drivers of tumor metastasis, the effects of baicalein on the metastatic ability of C918 and OCM-1 cells were assessed using scratch assay, transwell assay and detection of changes in EMT-related proteins. After baicalein treatment, the horizontal migration distance and the number of cells crossing the transwell membrane were significantly reduced (Figures 4A–G). In addition, the epithelial marker E-cadherin protein level was significantly increased and the mesenchymal marker N-cadherin with vimentin protein level was significantly decreased in C918 and OCM-1 cells (Figures 4H–M). Collectively, these findings suggest that baicalein suppresses the migration and EMT process of C918 and OCM-1 cells, thus exerting its anti-tumor effect.

### 3.5 Transcriptomic sequencing and Western blotting analysis of BAI treatment on CM cells

**FIGURE 5:**
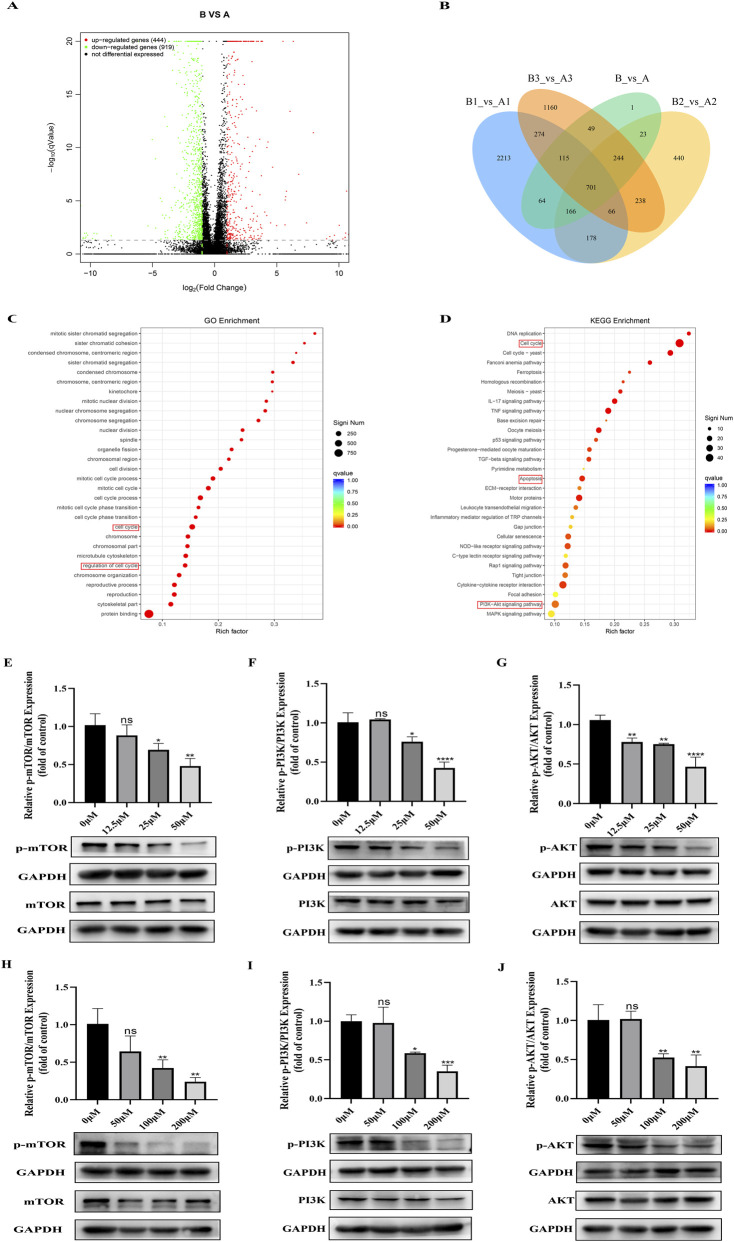
Transcriptomic sequencing and Western blotting analysis of BAI Treatment on CM cells. **(A)** Volcano plot illustrating DEGs. **(B)** Intersection of DEGs in each group. **(C,D)** Bubble map of GO and KEGG enrichment analysis. **(E–J)** Western blot analysis of changes in protein levels of p-mTOR, mTOR, p-AKT, AKT and p-PI3K, PI3K in C918 and OCM-1 cells after the action of different concentrations of BAI were presented in the form of histograms. Data are expressed as mean ± SEM. n = 3, ns: not significant, *P < 0.05, **P < 0.01, ***P < 0.001, ****P < 0.0001 vs. control.

To investigate the mechanism of baicalein against choroidal melanoma, we extracted the RNA of C918 cells from control and BAI groups after 48 h treatment and performed transcriptome sequencing analysis. As detailed in Supplementary Material Table S2, all sequencing libraries exhibited Q30 values exceeding 96.58%, indicating high base quality, thus ensuring the accuracy and reliability of our subsequent analysis of the sequencing results. A total of 1,333 differentially expressed genes were identified using P < 0.05 and |log2FoldChange| ≥ 1 as thresholds, of which 414 were upregulated genes and 919 were downregulated genes (Figures 5A,B). Functional enrichment analyses using GO terminology revealed significant biological process involvement of these DEGs in cell cycle regulation (Figure 5C). In addition, KEGG pathway enrichment analysis further revealed that the effects of BAI on choroidal melanoma were mainly focused on the cell cycle, apoptotic pathway and PI3K-AKT signaling pathway, which was consistent with our initial experimental hypothesis and network pharmacological predictions (Figure 5D). To establish mechanistic linkage between observed transcriptomic changes and PI3K/AKT/mTOR pathway modulation, we examined the changes in the expression of relative pathway proteins in C918 and OCM-1 cells using Western blot analysis. The results showed that the protein levels of p-PI3K, p-AKT and p-mTOR were significantly decreased after baicalein treatment (Figures 5E–J). This phosphorylation-specific inhibition provides direct molecular evidence supporting the hypothesis that BAI exerts its antiproliferative effects through disruption of this oncogenic pathway.

### 3.6 BAI suppressed oncogenic properties of CM cells by blocking the PI3K/AKT/mTOR axis

**FIGURE 6:**
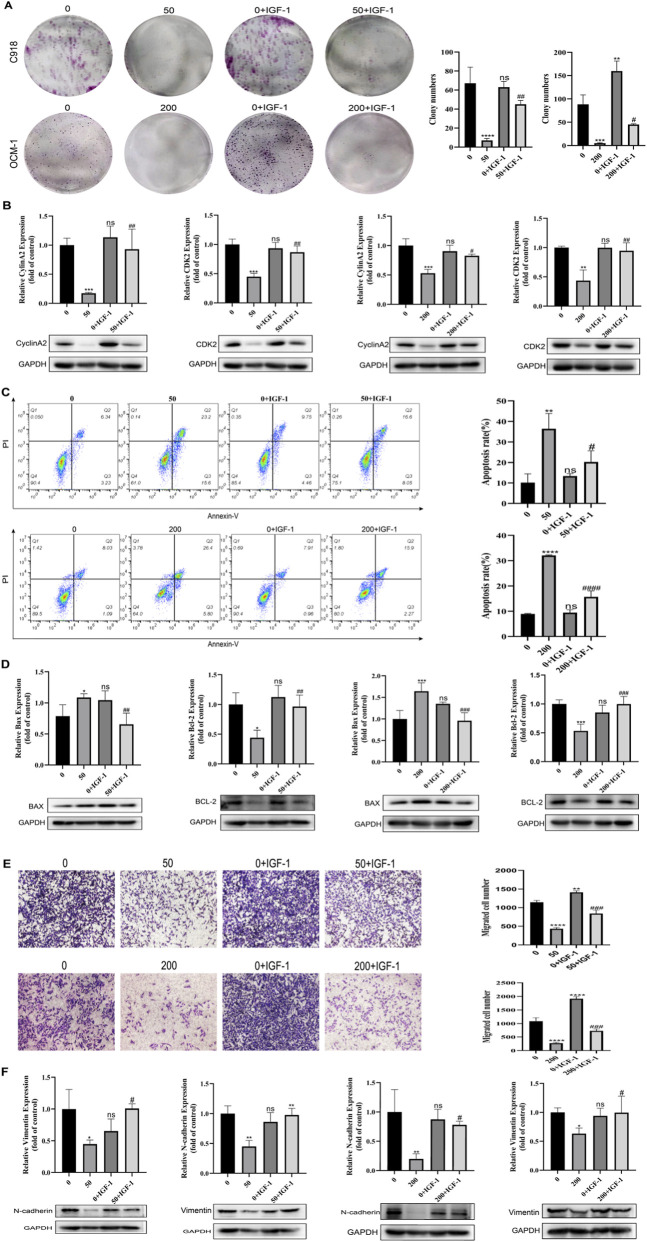
BAI suppressed oncogenic properties of CM cells by blocking the PI3K/AKT/mTOR axis. **(A)** Clone formation results of C918 cells and OCM-1 cells in the 0 μmol/L group, BAI alone dosing group, 0 μmol/L + IGF-1 group, and BAI + IGF-1 combination group. **(B)** Western blot analysis of the expression changes of CyclinA2 and CDK2. **(C)** Flow cytometry was used to detect and quantify the apoptosis rate C918 cells and OCM-1 cells. **(D)** Western blot analysis of the changes in the expression of Bax and Bcl-2 and grey scale value analysis. **(E)** Transwell assay was performed to detect the migration ability. **(F)** Western blot analysis of the changes in the expression of N-cadherin and Vimentin. Data are expressed as mean ± SEM. n = 3, ns: not significant, *P < 0.05, **P < 0.01, ***P < 0.001, ****P < 0.0001 vs. control group, #P < 0.05, ##P < 0.01, ###P < 0.001 vs. BAI alone dosing group.

To verify whether BAI could affect the biological behaviors of CM cells by targeting the PI3K/AKT/mTOR pathway, we designed co-treatment experiments using 200 ng/mL IGF-1 as a pharmacological activator of the PI3K/AKT/mTOR axis. Consistent with its established role in signaling activation (Supplementary Material Figures S1A–F), IGF-1 administration significantly enhanced phosphorylation levels across key nodes of this pathway. The outcomes of clone formation assay and the assessment of cell cycle proteins demonstrated that IGF-1 intervention markedly diminished the inhibitory impact of BAI on CM cell proliferation (Figures 6A,B). In addition, flow cytometry results showed that the apoptosis rate of IGF-1-treated cells was significantly decreased compared with that of BAI group, accompanied by an increase in the expression of the pro-apoptotic protein Bax and a decrease in the expression of Bcl-2, suggesting that IGF-1 could partially reverse BAI-induced apoptosis (Figures 6C,D). The findings from the scratch wound healing (Supplementary Material Figures S2A–D), transwell assay and the examination of EMT-related proteins found that IGF-1 could weaken the inhibitory effect of BAI on cell migration and EMT process (Figures 6E,F). Collectively, the attenuation of BAI’s antitumor effects by PI3K pathway activation supports our inference that BAI primarily exerts its oncosuppressive functions through targeted inhibition of this pathway.

### 3.7 BAI inhibits choroidal melanoma growth *in vivo*

**FIGURE 7:**
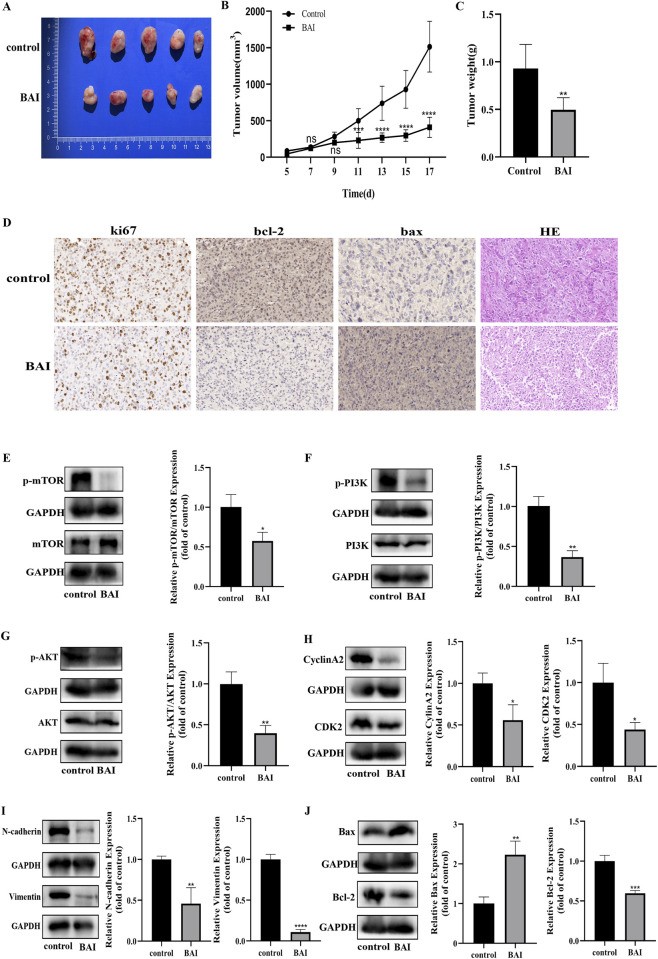
BAI inhibits choroidal melanoma growth *in vivo*. **(A–C)** Nude mice were executed 17 days after tumor seeding to measure the weight and volume of transplanted tumors. **(D)** Immunohistochemical analysis of Ki-67, Bax, Bcl-2 expression and observation of HE staining results. **(E–G)** Western blot to detect the expression changes of p-PI3K/PI3K, p-AKT/AKT and p-mTOR/mTOR in tumor tissues and grey scale value analysis. **(H–J)** Western blot detection of changes in expression of cycle, apoptosis and EMT-related proteins in tumor tissues and grey value analysis. Data are expressed as mean ± SEM. n = 5, ns: not significant, *P < 0.05, **P < 0.01, ***P < 0.001, ****P < 0.0001 vs. control.

Based on the results of the previous *in vitro* experiments and to determine whether BAI has a similar effect *in vivo*, we employed the C918 xenograft tumor model to evaluate the *in vivo* antitumor efficacy of BAI. As illustrated in Figure 7A, the growth rate of the transplant tumors in the BAI group was significantly slowed down compared with the control group. Furthermore, as evidenced by Figures 7B,C, BAI treatment resulted in a substantial reduction in both the weight and volume of the C918 xenografted tumors without causing significant changes in mice body weight (Supplementary Material Table S4). In addition, HE staining analysis demonstrated morphological alterations in BAI-treated tumors, characterized by expanded interstitial matrix and diminished malignant features (Figure 7D). Immunohistochemical staining further revealed a significant suppression of Ki-67 and Bcl-2 expression, accompanied by an elevation in Bax expression. To clarify the molecular mechanisms underlying BAI’s therapeutic action, changes in PI3K/AKT/mTOR signaling pathway proteins, apoptosis, cycle and migration related proteins were detected using Western blot. The findings revealed that BAI treatment significantly suppressed the expression of p-PI3K/PI3K, p-AKT/AKT and p-mTOR/mTOR. Additionally, the expression levels of Bax, Bcl-2, CyclinA2, CDK2, Vimentin, and N-cadherin were also decreased in the BAI-treated group (Figures 7E–J). In conclusion, these results indicate that BAI can inhibit the growth of choroidal melanoma *in vivo*, thereby providing a rationale for its potential therapeutic application in the management of this malignancy.

## 4 Discussion

The pathogenesis of choroidal melanoma is complex and regulated by multiple molecular and cellular signaling pathways. In recent years, the potential of natural products in the field of tumor intervention has attracted widespread attention, among which Scutellaria baicalensis, family Labiatae, has become a research hotspot due to its multi-targeted anti-tumor properties. However, the specific efficacy and underlying mechanisms of its active component, baicalein, in CM remain largely unexplored. Unlike previous studies that relied on single-dimensional analyses or computational predictions alone, our study provides the first comprehensive and systematic validation of baicalein’s anti-CM effects by integrating network pharmacology, transcriptomic profiling, and rigorous vitro and *in vivo* functional assays. By integrating *in silico* predictions with experimental validation, we have successfully deciphered the molecular mechanism of baicalein and provided a solid preclinical rationale for its therapeutic application in CM. This comprehensive multi-omics strategy sets our study apart from mere pathway verification, presenting a novel framework for drug repositioning in this rare yet fatal malignancy.

In this study, we first used a multi-database cross-validation strategy to obtain 106 baicalein action targets from the TCSMP platform, and compared and analyzed them with CM-related targets in Genecards, PharmGkb, Disgenet and Omim databases, and finally screened 41 potential action targets. Based on the construction of STRING protein interaction network and the identification of core modules in Cytoscape 3.9.1, we found that 10 hub genes, including AKT1, IL-6, TP53, etc., constituted the key regulatory network. GO and KEGG enrichment analyses further revealed that the mitochondrial apoptosis pathway and the PI3K/AKT signaling cascade are the core pathways through which baicalein exerts its anti-tumor effects. These bioinformatics predictions provide direction for subsequent mechanistic studies.

Based on the predicted outcomes derived from network pharmacology analysis, the C918 and OCM-1 cell lines were chosen as the subjects for functional validation in this study. CCK8 assay established the effective intervention concentration of the 2 cell lines, confirming that baicalein significantly inhibited the proliferative activity of the cells. Notably, the 2 cell lines exhibited distinct drug sensitivities, a phenomenon characterized by heterogeneity, which is frequently encountered in tumor pharmacology research. A comparable observation was reported in the study conducted by Zhang et al., where MG63 and 143B cells displayed varying sensitivities to baicalein, yet both were effectively suppressed by this compound (Wen et al., 2023). Beyond direct proliferation suppression, modulation of cell cycle has emerged as a critical therapeutic strategy in oncology. The progression of the cell cycle directly governs the rate of cell division and growth, and its aberrant regulation is intimately associated with tumor development. Post-treatment analyses demonstrated marked S-phase arrest in both cell lines, accompanied by downregulation of key regulatory proteins including Cyclin A2 and CDK2. This finding aligns with the mechanism proposed by Liu et al. in their cervical cancer study, which revealed that baicalein induces cell cycle arrest by inhibiting the PIM1/CDK2 axis (Wang et al., 2025). Interestingly, the differential response of the 2 cell lines to baicalein may be related to their intrinsic biological properties. For example, the C918 cell line has a more aggressive profile, whereas the OCM-1 cell line exhibits a relatively slow proliferation rate, and this heterogeneity may lead to differences in the selectivity of drug targets. Despite the cell line-specific response, baicalein was able to achieve proliferation inhibition by inducing S-phase blockade in both cell lines, suggesting that cell cycle regulation may be one of the core pathways for its antitumor effect.

Programmed cell death (PCD), particularly the mitochondria-mediated intrinsic apoptotic pathway, is a key regulator of tumor cell survival. This pathway is mainly controlled by the Bcl-2 family of proteins, where the balance between pro-apoptotic proteins (such as Bax) and anti-apoptotic proteins (such as Bcl-2) determines cell fate (Yang et al., 2019; Cheng et al., 2018). Our study found that treatment with baicalein significantly disrupted this balance, manifested by upregulation of Bax and downregulation of Bcl-2, thereby inducing increased mitochondrial outer membrane permeability. These observations are consistent with previous reports that baicalein induces mitochondrial apoptosis in other tumor models, confirming the central role of the mitochondrial apoptotic pathway in baicalein’s anti-CM effects.

Epithelial-mesenchymal transition (EMT) is a key biological process that endows cancer cells with migratory and invasive abilities and is also an early event in tumor metastasis (Chen et al., 2019; Friend et al., 2023). Although choroidal melanoma originates from neural crest cells, it often exhibits EMT-like phenotypic characteristics during malignant progression. Prior investigations have elucidated that the transcription factor PRRX1 and abnormal activation of the Wnt/β-catenin pathway can significantly promote the metastasis of uveal melanoma (Meng et al., 2022; Zheng and Pan, 2018). Based on these findings, targeted intervention of the EMT signaling axis and key regulatory factors may become an effective therapeutic strategy to inhibit choroidal melanoma progression. Consistent with prior research, our study reveals that baicalein significantly attenuates the migratory capacity of C918 and OCM-1 CM cell lines. Mechanistically, this inhibitory effect correlates with the downregulation of N-cadherin and vimentin expression, accompanied by the upregulation of E-cadherin levels. These findings provide direct experimental evidence that baicalein inhibits choroidal melanoma cell migration by regulating the EMT pathway, expanding the prospects for the application of TCM components in tumor therapy.

To further delineate the underlying molecular events, we conducted gene expression profiling using transcriptome sequencing technology in this study. The sequencing results showed that the differentially expressed genes in the baicalein-treated group were significantly enriched in the cell cycle regulation, apoptosis execution, and PI3K/AKT signaling pathways, which was highly consistent with the network pharmacology prediction. The PI3K/AKT/mTOR pathway, a pivotal intracellular signaling network, governs cellular processes such as growth, motility, metabolism, and angiogenesis (Katso et al., 2001; Martini et al., 2014). and its aberrant activation is closely associated with the malignant progression of CM (Chen et al., 2022; Shi et al., 2021b). Our further studies demonstrated that baicalein significantly suppressed the phosphorylation of key components in this pathway, accompanied by altered expression of downstream cell cycle-regulatory proteins, EMT-related markers, and apoptotic regulators. Notably, these effects were partially abrogated by the pathway-specific agonist IGF-1, directly confirming the pathway’s specific mediating role in baicalein’s pharmacological mechanism.

At the *in vivo* level, a xenograft tumor model in nude mice showed that baicalein significantly inhibited tumor growth, reducing tumor volume and weight, without displaying obvious systemic toxicity. Immunohistochemistry and Western blot analyses confirmed that the expression changes of PI3K/AKT/mTOR pathway components and their downstream effectors in in vivo experiments were consistent with the *in vitro* observations. These data strongly support the core conclusion that “baicalein suppresses choroidal melanoma progression by targeting the PI3K/AKT/mTOR signaling axis.

Although the current study underscores the therapeutic promise of baicalein in choroidal melanoma, certain limitations still exist. First, in studies of network pharmacology, we did not use filtering strategies beyond STRING/Cytoscape topology or experimentally validate all the core genes, which may lead to false positives. Second, the action network of baicalein is extremely complex. In addition to the PI3K/AKT pathway, previous investigations in other disease models have demonstrated that baicalein modulates multiple signaling pathways, including the JAK/STAT pathway (Ke et al., 2019), the Nrf2/Keap1 axis (Li et al., 2020), and the MAPK signaling cascade (Wang et al., 2010). While this study focused on the PI3K/AKT axis, the synergistic effects of other pathways or non-coding RNA regulatory networks cannot be ruled out. Third, while the xenograft data supports the anti-tumor potential of baicalein, the use of a single dose and cell line prevents the establishment of a dose-response relationship. Furthermore, comprehensive toxicity assessments and pharmacokinetic profiling were not performed in this study. Consequently, the translational application of these findings requires extensive validation, including dose-ranging studies, PK/PD analysis, and GLP-compliant toxicity testing in the future. More importantly, the clinical translation of baicalein still faces pharmacokinetic challenges, such as low oral bioavailability and a short half-life (Yu et al., 2017b). Therefore, future research should focus on developing novel drug delivery systems to improve its bioavailability and conduct rigorous preclinical pharmacokinetic and safety evaluations, thereby promoting the translation of baicalein into a clinical anti-tumor drug.

## 5 Conclusion

Taken together, our data demonstrate for the first time that BAI inhibits choroidal melanoma progression primarily by modulating the PI3K/AKT/mTOR signaling pathway without inducing significant cytotoxicity. Although additional studies are necessary to thoroughly evaluate its safety profile and clinical efficacy, these findings provide novel insights and a promising direction for the discovery and development of new therapeutic agents targeting choroidal melanoma.

## Data Availability

The data presented in the study are deposited in the Figshare repository, accession number 10.6084/m9.figshare.31883326.

## Supplementary material

SUPPLEMENTARY FIGURE S1 Western blot analysis was used to validate the activation of the PI3K/AKT/mTOR pathway by IGF-1.

SUPPLEMENTARY FIGURE S2 The scratch assay was used to detect the effect of IGF-1 on cell migration ability.
