# Supplementary figures and images for: Exploring the mechanism of baicalein on choroid melanoma based on network pharmacology, transcriptomics and experimental verification
Source: Front Pharmacol. 2026 Apr 8;17:1787798. doi: 10.3389/fphar.2026.1787798 (PMC13099128; doi:10.3389/fphar.2026.1787798)

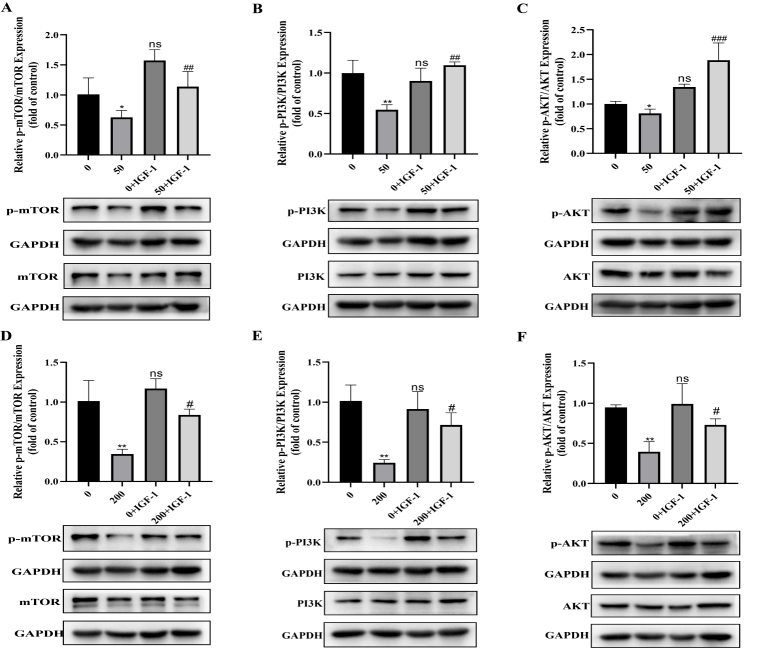

Supplement: Supplementary file 1 [file Image1.tiff]

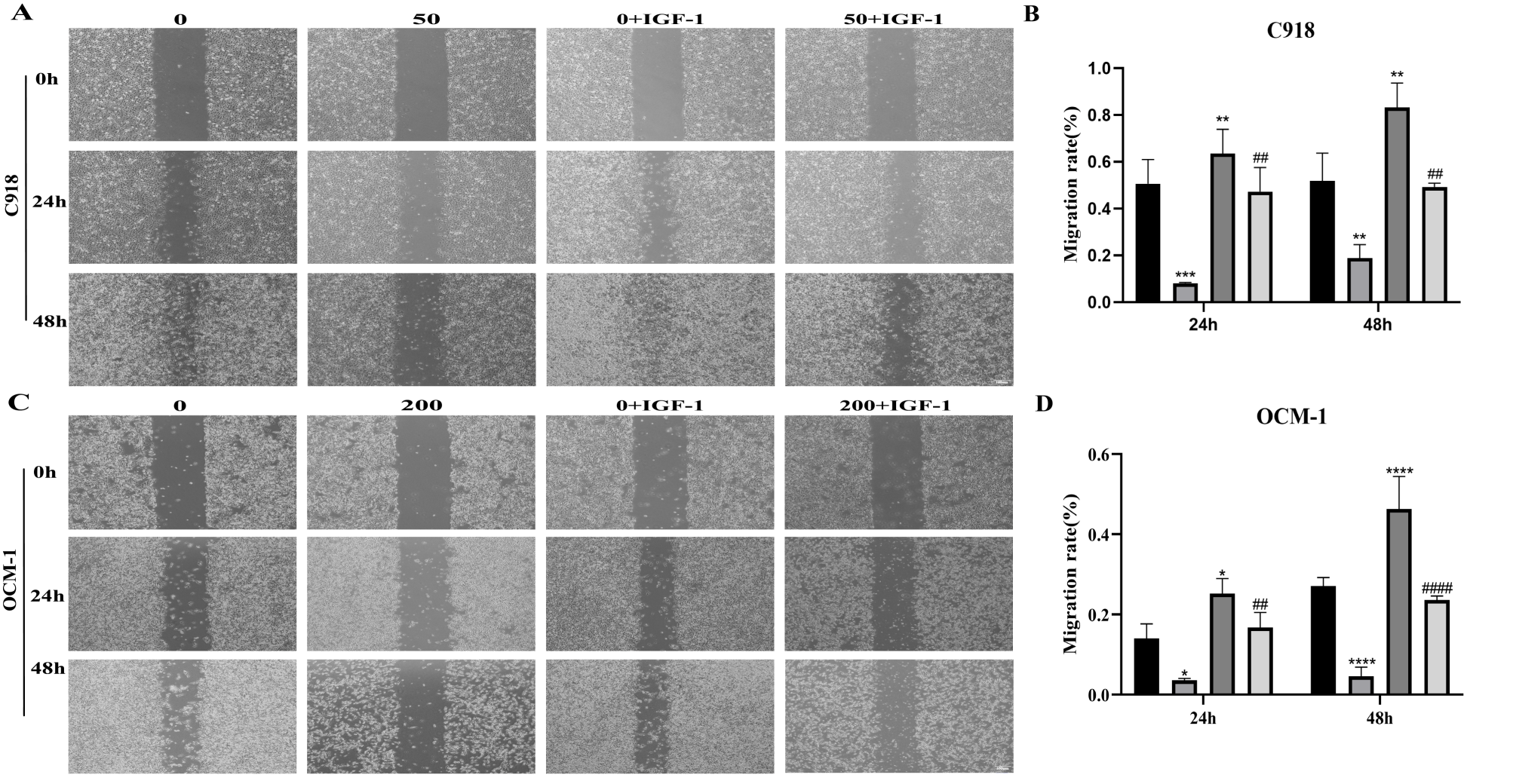

Supplement: Supplementary file 6 [file Image2.tiff]
